# Pharmacokinetics of minocycline with and without rifampicin co-administration in patients with nontuberculous mycobacterial disease

**DOI:** 10.1093/jac/dkag262

**Published:** 2026-07-30

**Authors:** Arthur Lemson, Rob Aarnoutse, Lisa Houtman, Rob Arts, Arjan van Laarhoven, Jakko van Ingen, Wouter Hoefsloot, Ralf Stemkens

**Affiliations:** Department of Pulmonary Diseases, Research Institute for Medical Innovation, Radboudumc Community for Infectious Diseases, Radboud University Medical Center, Nijmegen, The Netherlands; Department of Pharmacy, Pharmacology & Toxicology, Research Institute for Medical Innovation, Radboudumc Community for Infectious Diseases, Radboud University Medical Center, Nijmegen, The Netherlands; Department of Pulmonary Diseases, Research Institute for Medical Innovation, Radboudumc Community for Infectious Diseases, Radboud University Medical Center, Nijmegen, The Netherlands; Department of Internal Medicine, Research Institute for Medical Innovation, Radboudumc Community for Infectious Diseases, Radboud University Medical Center, Nijmegen, The Netherlands; Department of Internal Medicine, Research Institute for Medical Innovation, Radboudumc Community for Infectious Diseases, Radboud University Medical Center, Nijmegen, The Netherlands; Department of Medical Microbiology, Research Institute for Medical Innovation, Radboudumc Community for Infectious Diseases, Radboud University Medical Center, Nijmegen, The Netherlands; Department of Pulmonary Diseases, Research Institute for Medical Innovation, Radboudumc Community for Infectious Diseases, Radboud University Medical Center, Nijmegen, The Netherlands; Department of Pharmacy, Pharmacology & Toxicology, Research Institute for Medical Innovation, Radboudumc Community for Infectious Diseases, Radboud University Medical Center, Nijmegen, The Netherlands

## Abstract

**Objectives:**

Minocycline demonstrates *in vitro* activity against *Mycobacterium avium* complex (MAC), but pharmacokinetic data in patients are limited. Additionally, co-administration with rifampicin may significantly alter minocycline pharmacokinetics. We evaluated the pharmacokinetics of minocycline with and without rifampicin co-administration in patients with nontuberculous mycobacterial (NTM) disease.

**Methods:**

This one-arm, two-period, fixed-order study was conducted at Radboud University Medical Center in the Netherlands. Adults with NTM disease eligible for rifampicin-based therapy received two 5-day courses of minocycline 200 mg once daily, before and a month after initiating rifampicin in a once-daily dose of 450 mg (<50 kg) or 600 mg (≥50 kg). Minocycline pharmacokinetics were assessed over 24 h on Day 5 during both courses, i.e. at steady state. Rifampicin concentrations were evaluated concurrently during the second course. Non-compartmental and bioequivalence analyses were performed.

**Results:**

Eleven patients completed the first minocycline course; nine completed both. Geometric mean values (95% CI) of the total minocycline exposure (AUC_0–24_), peak concentration and pre-dose concentration without rifampicin co-administration were 58.4 mg·h/L (48.7–70.1), 5.2 mg/L (4.2–6.3) and 1.0 mg/L (0.8–1.3), respectively. The geometric mean ratio for the AUC_0–24h_ of minocycline with versus without rifampicin was 49% (90% CI, 44.1–54.4), below the standard bioequivalence range of 80%–125%. All adverse events were graded mild or moderate. Nine (13%) were attributed to minocycline, primarily gastrointestinal, vestibular and headache-related.

**Conclusions:**

This study provides data on minocycline pharmacokinetics in patients with NTM disease. Co-administration with rifampicin resulted in a halving of minocycline exposure, likely due to induction of drug-metabolizing enzymes and/or transporters.

## Introduction

Nontuberculous mycobacterial (NTM) disease is emerging as an important opportunistic infection of humans.^1^ Pulmonary disease (PD) by *Mycobacterium avium* complex (MAC) bacteria is the most common manifestation, with suboptimal treatment outcomes despite prolonged multidrug therapy,^2^ partly due to suboptimal dosing strategies, drug–drug interactions and intolerability.^3,4^ For MAC disease, the current guideline recommends a treatment regimen that includes azithromycin, ethambutol and rifampicin.^3^ The need for drug substitutions is common, yet the use of alternative efficacious therapies is often restricted by adverse effects, cost and limited accessibility.^5,6^

Minocycline, a tetracycline antibiotic, shows promise as it is active *in vitro* against multiple mycobacterial species, including MAC.^7–10^ In hollow fibre models, minocycline exerts a concentration-dependent effect against MAC and provides additional benefit when used in combination with azithromycin and ethambutol.^9,11^ Moreover, an observational study of patients with MAC-PD has shown that a regimen combining clarithromycin, clofazimine and minocycline yields outcomes comparable to those achieved with guideline-recommended treatment.^12^ Common adverse effects of minocycline involve disturbances of the gastrointestinal, hepatobiliary, nervous, musculoskeletal, cutaneous and immune systems.^13^ Little data are available regarding the association between exposure metrics of minocycline and toxicity. Long-term use of minocycline has been proven safe and relatively tolerable, although the risk of autoimmune reactions such as systemic lupus erythematosus increases with prolonged exposure, and high doses (up to 200 mg per day) are associated with an increased risk of skin pigmentation.^14–17^

Pharmacokinetic (PK) data on minocycline in a population with NTM disease are not available. Additionally, there are no data on minocycline co-administration with rifampicin, a well-known inducer of metabolic enzymes and drug transporters, which reduces the exposure to another tetracycline, doxycycline.^18,19^ This study aimed to (i) describe the pharmacokinetics of minocycline and (ii) evaluate the effect of rifampicin on the pharmacokinetics of minocycline in patients with NTM disease.

## Patients and methods

### Study population

We enrolled adults diagnosed with NTM disease, eligible for a rifampicin-based regimen (in- and exclusion criteria in Table S1 (available as Supplementary data at *JAC* Online)).^3^ In NTM disease, the AUC is considered to be the most important exposure parameter for the efficacy of minocycline.^20,21^ A sample size of 12 participants was calculated to estimate the area under the concentration versus time curve from T = 0 up to T = 24 h (AUC_0–24h_) of minocycline at steady state, with 15% precision (margin of error) at a two-sided α of 0.05. This calculation was based on a previously reported AUC from zero to infinity of 19.6 mg·h/L after a single dose of 100 mg and a standard deviation of 4.4 mg·h/L.^22^ Due to differences between the reference study and our study (e.g. study population, drug formulation, analytical method for measurement of minocycline concentration), we incorporated a safety margin of three additional patients, leading to a definitive intended sample size of 15. Written informed consent was obtained from all participants prior to enrolment. The study (clinicaltrials.gov trial number: NCT05861258) was approved by the institutional review board METC Oost-Nederland (reference number: NL69313.091.19) and conducted in compliance with the principles of Good Clinical Practice.

### Study design

**Figure 1.**
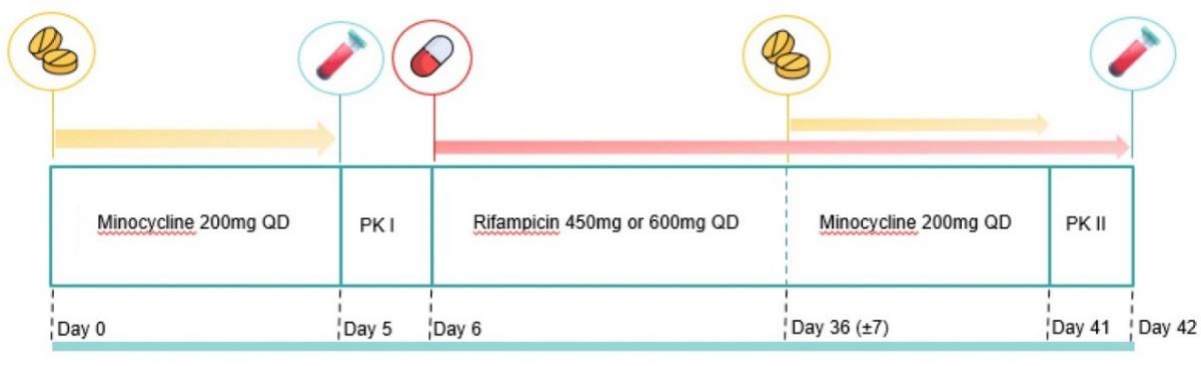
Overview of the study design. Two 5-day courses of minocycline 200 mg once daily were administered. The first course was completed prior to starting rifampicin. The second course was administered with rifampicin co-administration, 1 month (±1 week) after starting rifampicin. On Day 5 of both courses (i.e. PK I and PK II), minocycline plasma concentrations were determined at T = 0 (pre-dose), 1, 2, 3, 4, 6, 8 and 24 h after administration. In addition, rifampicin plasma concentrations were determined at T = 2, 4 and 6 h after administration at PK visit II.

This was an open-label, one-arm, two-period, fixed-order pharmacokinetic study conducted at Radboud University Medical Center (Radboudumc), Nijmegen, the Netherlands. The primary objective was to describe the pharmacokinetics of minocycline in patients with NTM disease during steady-state conditions. The secondary objective was to evaluate the effect of rifampicin on the pharmacokinetics of minocycline. All patients received two 5-day oral courses of minocycline (brand Aurobindo) 200 mg once daily.^23^ The first course was administered prior to the initiation of rifampicin, and the second course started after 1 month (±1 week) of rifampicin treatment (Figure 1). Oral rifampicin was dosed based on body weight (450 mg once daily if <50 kg, 600 mg once daily if ≥50 kg). A 5-day course was selected to ensure steady-state (stable) concentrations of minocycline at the time of sampling.^24^ The interval between courses was selected to allow sufficient time for complete induction of metabolic enzymes and transporters by rifampicin and to achieve full auto-induction and thus steady state for rifampicin.^25,26^

On Day 5 of each course, minocycline was administered on an empty stomach after overnight fasting. Blood samples were collected in lithium-heparin tubes (without gel separation layer) at 8 consecutive time points for measurement of minocycline plasma concentrations at 0 (pre-dose), 1, 2, 3, 4, 6, 8 and 24 h after administration. During the second course, samples taken at 2, 4 and 6 h post-administration were also used to measure rifampicin plasma concentrations. Total plasma concentrations were measured using validated ultra-performance liquid chromatography (minocycline) and liquid chromatography–mass spectrometry methods (rifampicin) (details in Table S2). The calibration ranges were 0.025–20.0 mg/L for minocycline and 0.09–60.0 mg/L for rifampicin. Assay accuracies ranged from 89% to 103% (intraday) and 91% to 102% (interday) for minocycline and 96% to 103% (intraday) and 96% to 101% (interday) for rifampicin. Intraday precision was 1.5%–2.8% for minocycline and 2.6%–4.9% for rifampicin, and interday precision was 0.0%–2.3% and 0.0%–1.9%, respectively.

Adherence to both drugs was monitored through intake diaries and self-reporting, along with additional pill counts for minocycline. Antimycobacterial drugs other than rifampicin were started prior to or together with the first minocycline course, in accordance with international guidelines.^3^ Furthermore, changes in concomitant medications during the study were reviewed to identify potential drug–drug interactions with minocycline.

### Non-compartmental PK analysis

Non-compartmental analysis was performed using Phoenix WinNonlin v8.6 (Certara USA Inc., Princeton, NJ, USA) to determine PK parameters of minocycline. These included the area under the plasma concentration versus time curve (AUC_0–24h_), peak concentration (C_max_), time to C_max_ (T_max_), pre-dose (trough) concentration (C_trough_), apparent volume of distribution (Vd/F, where F is bioavailability), apparent clearance (Cl/F) and elimination half-life (T_1/2_) of minocycline. The AUC_0–24h_ of rifampicin was estimated based on concentrations measured at T = 2, 4 and 6 h after administration, using a limited sampling formula derived from a study in a tuberculosis population (AUC_0–24_ = −2.22 + 2.05 × *C*_2_ + 2.25 × *C*_4_ + 4.93 × *C*_6_).^27^ Rifampicin C_max_ was the highest measured concentration among these three samples, and T_max_ was the time to C_max_.

### Bioequivalence analysis

A bioequivalence analysis on all PK data with a linear mixed-effects model was performed to assess the effect of rifampicin on the exposure to minocycline. The geometric mean ratios (GMRs) of the AUC_0–24h_, C_max_ and C_trough_ of minocycline (with versus without rifampicin) were calculated. A GMR estimate with a 90% CI completely within the standard bioequivalence range of 80%–125% was predefined as no significant drug–drug interaction.^28^

### Safety and tolerability

Patients were monitored for the frequency and severity of adverse events (AEs) during and between minocycline courses, using daily diaries, structured recording of medical histories and safety laboratory testing. AEs were graded using the Common Terminology Criteria for Adverse Events (CTCAE, v.5.0).^29^

### Statistical analysis

Descriptive statistics were performed with SPSS version 29. Categorical data were presented as counts and proportions, while continuous data were summarized as median with interquartile range (25th–75th percentile). PK parameters were reported as GMs with their corresponding 95% CIs and minimum and maximum values. The AUC_0–24h_, C_max_ and C_trough_ ratios of minocycline (i.e. with versus without rifampicin) were correlated with the total exposure (AUC_0–24h_) to rifampicin using Spearman rank correlation.

## Results

### Study population

The intended 15 participants could not be reached as a result of enrolment difficulties (e.g. less use of rifampicin for MAC disease at the study site). Twelve patients were enrolled, of whom nine completed the study. One participant withdrew during and two after the first minocycline course (details in Table S3). Among 11 patients who completed the first course, 4 (36.4%) were female, and the median age was 61 years (IQR, 52–67) (Table 1). The majority of patients (10 out of 11) were diagnosed with NTM-PD. Baseline characteristics showed no numerical differences when correcting for the two early withdrawals (Table 1).

**Table 1.** Study population

	Minocycline course I completed	Minocycline course I and II completed
*N*	11^a^	9^b^
Age (years), median (IQR)	61 (52–67)	64 (53–60)
Female sex, *n* (%)	4 (36)	3 (33)
Body weight (kg), median (IQR)	62.8 (53–65.6)	64.3 (56.5–65.9)
Body mass index (kg/m^2^), median (IQR)	19.6 (18.3–21.2)	19.6 (18.7–22.2)
Ethnicity, Caucasian, *n* (%)	11 (100)	9 (100)
Pulmonary disease, *n* (%)	10 (91)	8 (89)
Extrapulmonary disease, *n* (%)	1 (9)	1 (11)
Active smokers, *n* (%)	1 (9)	1 (11)
Other antimycobacterial drugs used, *n* (%)		
Azithromycin (%)	10 (91)	8 (89)
Ethambutol (%)	11 (100)	8 (89)
Clofazimine (%)	5 (46)	4 (44)
Amikacin (parenteral) (%)	7 (64)	4 (44)
Isoniazid (%)	1 (9)	1 (11)

^a^One participant withdrew before completing the first minocycline course and was therefore excluded from this analysis.

^b^Two participants withdrew after completing the first minocycline course.

### Non-compartmental analysis and descriptive PK

**Figure 2.**
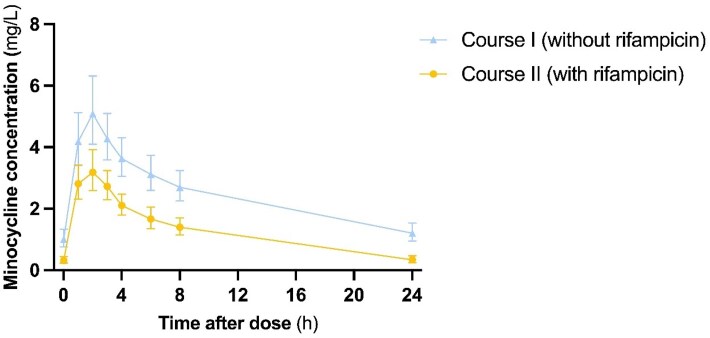
Minocycline plasma concentration profiles with and without rifampicin co-administration. Geometric mean (95% CI) minocycline plasma concentration versus time profile of course 1 (without rifampicin) and course 2 (with rifampicin). Dots and triangles represent geometric means per sample time; vertical error bars represent the corresponding 95% CIs.

**Figure 3.**
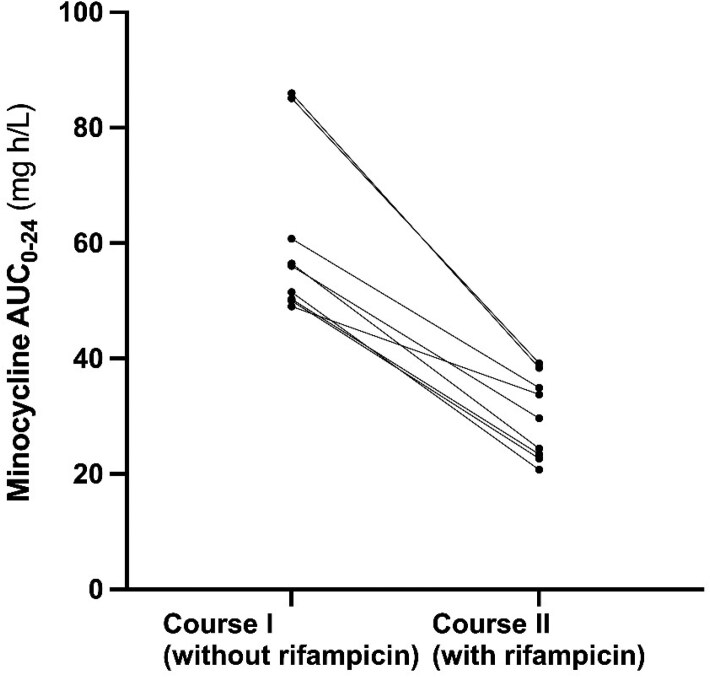
Individual minocycline total plasma exposures before and during co-administration of rifampicin. AUC_0–24h_, area under the plasma concentration versus time curve from 0 to 24 h.

A total of 160 minocycline samples were analysed for 11 patients across two study periods, none of which were excluded or fell below the lower limit of quantification. The geometric mean (95% CI) AUC_0–24h_, C_max_ and C_trough_ of minocycline without rifampicin co-administration were 58.4 mg·h/L (48.7–70.1), 5.2 mg/L (4.2–6.3) and 1.0 mg/L (0.8–1.3), respectively (Table 2). The observed ranges were 36.4–86.0 mg·h/L, 3.5–9.2 mg/L and 0.5–1.9 mg/L. Interpatient variability, expressed as coefficient of variation (CV%), was 27.6%, 31.5% and 44.5% for AUC_0–24h_, C_max_ and C_trough_, respectively. Geometric mean AUC_0–24h_, C_max_ and C_trough_ of minocycline during rifampicin co-administration were lower: 28.9 mg·h/L, 3.4 mg/L and 0.3 mg/L, respectively (Figures 2 and 3). AUC_0–24h_ decreased in all nine patients, whereas C_max_ was reduced in eight out of nine individuals. The geometric mean (95% CI) AUC_0–24h_ and C_max_ of rifampicin were 53.8 mg·h/L (33.9–85.4) and 12.3 mg/L (8.4–18.1), respectively, with a median T_max_ of 2.0 h (IQR, 2.0–2.1). There was no significant correlation between the rifampicin AUC_0–24h_ and the extent of the effect of rifampicin on the exposure to minocycline (Spearman’s rho = 0.03, *P* = 0.93 for AUC_0–24h_ of minocycline; Spearman’s rho = 0.28, *P* = 0.46 for C_max_ of minocycline; Spearman’s rho = −0.07, *P* = 0.87 for C_trough_).

**Table 2.** Pharmacokinetics of minocycline and rifampicin

PK parameter	Minocycline without rifampicin (*N* = 11)		Minocycline with rifampicin (*N* = 9)	
	GM (95% CI)	CV%	GM (95% CI)	CV%
AUC_0–24h_ (mg·h/L)	58.4 (48.7–70.1)	27.6	28.9 (24.0–34.9)	24.8
C_max_ (mg/L)	5.2 (4.2–6.3)	31.5	3.4 (3.0–4.0)	18.9
T_max_ (h), median (range)	2.0 (1.0–3.0)	28.6	2.0 (1.0–3.0)	31.3
C_trough_ (mg/L)	1.0 (0.8–1.3)	44.5	0.3 (0.2–0.4)	43.2
Vd/F (L)	65.7 (54–80)	29.9	78 (67.9–89.5)	18.2
Cl/F (L/h)	3.4 (2.9–4.1)	27.6	6.9 (5.7–8.3)	24.8
T_1/2_ (h)	13.3 (11.5–15.3)	21.4	7.8 (7.0–8.8)	15.2

AUC_0–24h_, area under the plasma concentration versus time curve from 0 to 24 h; Cl/F, apparent clearance; C_max_, peak concentration; C_trough_, pre-dose (trough) concentration; CV%, coefficient of variation; GM, geometric mean; PK, pharmacokinetic; T_1/2_, elimination half-life; T_max_, time to peak concentration; Vd/F, apparent volume of distribution.

### Bioequivalence analysis

**Figure 4.**
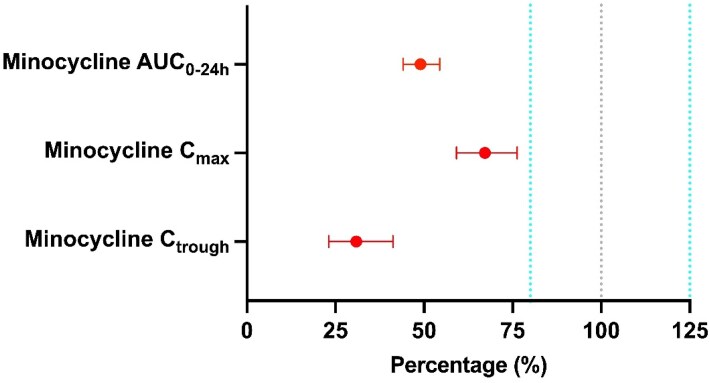
Results of the bioequivalence analysis (effect of rifampicin co-administration on the exposure to minocycline). AUC_0–24h_, area under the plasma concentration versus time curve from 0 to 24 h; C_max_, peak concentration; C_trough_, pre-dose concentration. The middle dashed line indicates the unity line at 100% (i.e. no difference in minocycline exposure with and without rifampicin co-administration). The outer dashed lines indicate the standard bioequivalence range of 80%–125% (i.e. absence of a significant drug-drug interaction).^28^ Dots and error bars reflect point estimates and 90% CIs of geometric mean ratios of minocycline exposures (with rifampicin versus without rifampicin).

GM ratios of minocycline exposure measures (with versus without rifampicin) were 49.0% (90% CI, 44.1–54.4) for AUC_0–24h_, 67.2% (90% CI, 59.2–76.2) for C_max_ and 30.9% (90% CI, 23.1–41.2) for C_trough_ (Figure 4). These ratios and their CIs fell entirely below the predefined bioequivalence range (80%–125%).

### Adverse events

Adverse events were common throughout the study: 69 were reported (Table 3). All were mild or moderate (CTCAE grade < 3), and 50 (72.5%) were deemed unlikely to be related to minocycline, 10 (14.5%) were possibly related, and 9 (13%) were considered related. Minocycline-related AEs included gastrointestinal disturbance, vestibular disorder, headache, anorexia and hyperhidrosis.

**Table 3.** Adverse events occurring during or between minocycline courses

*N*	12^a^
Total number of AEs	69
Total number of SAEs	0
CTCAE grade	
Grade I (%)	44 (63.8%)
Grade II (%)	25 (36.2%)
AEs occurring in ≥25% of patients (%)	
Gastrointestinal disturbances	16 (23.2%)
Ototoxicity	4 (5.8%)
Fatigue	4 (5.8%)
Discoloration of body fluid	4 (5.8%)
Anorexia	4 (5.8%)
Vestibular disorder	3 (4.3%)
Skin-related	3 (4.3%)
Headache	3 (4.3%)
Dysgeusia	3 (4.3%)
Relatedness to minocycline (%)	
Unlikely related	50 (72.5%)
Possible related	10 (14.5%)
Probably related	9 (13%)
Minocycline discontinued due to AE (%)	1 (8.3%)

^a^All participants were included in the AE analysis.

AE, adverse event; CTCAE, Common Terminology Criteria for Adverse Events; SAE, serious adverse event.

## Discussion

This study is the first to characterize the pharmacokinetics of minocycline in patients with NTM disease and to evaluate its interaction with rifampicin. At steady state, the geometric mean total exposure (AUC_0–24h_) of minocycline was 58.4 mg·h/L (95% CI, 48.7–70.1) with inter-individual variability that corresponded with previous studies on minocycline.^22,30^ Co-administration with rifampicin resulted in a 51% reduction in total exposure, indicating a drug–drug interaction, although this was based on a limited number of nine patients.^28^ The findings in this study offer population-level PK estimates for two treatment scenarios and may inform dosing strategies in future interventional studies.

The study cohort primarily comprised adults over 50 years of age with advanced NTM-PD requiring antimycobacterial therapy. Limited data are available on the PK of minocycline. The AUC is thought to be the most important exposure parameter for the efficacy of minocycline in NTM disease and other infections.^20,21^ In two healthy volunteer studies from the 1970s, mean AUCs from zero to infinity (pharmacologically equivalent to AUC_0–24h_ at steady state) of 71.1 and 71.3 mg·h/L (no ranges reported) were observed after a single 200 mg dose of minocycline.^31,32^ This was slightly higher than the GM AUC_0–24h_ of 58.4 mg·h/L in our study. The GM peak concentration in our study was 5.2 mg/L, whereas peak concentrations in the abovementioned studies were 3.1 mg/L (range 2.7–3.7) and 3.5 mg/L (no range reported), respectively.^31,32^ However, direct comparisons with our C_max_ are not appropriate, as our cohort received multiple doses, resulting in drug accumulation, and the earlier studies involved single-dose administration. In another study, minocycline PK was assessed after a multi-day course in patients undergoing elective surgery.^33^ The reported mean serum peak concentration was 3.5 mg/L (no range reported), but participants received a dose of 100 mg twice daily versus 200 mg once daily in our study. Moreover, only pre-dose and 3 h post-dose levels were measured versus extensive sampling in our study, which likely enabled us to better capture the peak concentration. A mean trough concentration was reported only in the multiple-dosing study, namely 2.3 mg/L (no range reported).^33^

The geometric mean ratios of the AUC_0–24h_, C_max_ and C_trough_ of minocycline (with versus without rifampicin) and accompanying 90% CIs were entirely below the standard bioequivalence range of 80%–125%, indicating a PK interaction (Figure 4). Also, the effect was consistent across the study population (Figure 3), which was reflected by relatively narrow CIs of the GMRs (Figure 4). Rifampicin co-administration reduced the AUC_0–24h_ of minocycline by 51%, which is considered a moderate interaction according to drug–drug interaction guidelines.^34,35^ However, the clinical relevance of this drug–drug interaction is unknown at this point. Minocycline undergoes partial hepatic biotransformation to mostly inactive metabolites, although the exact pathway remains unknown.^36–39^ Some metabolites of minocycline have very low antimicrobial activity and are unlikely to add significant therapeutic activity in MAC disease.^39^ Rifampicin is a strong inducer of drug-metabolizing enzymes and transporters, which likely explains the marked reductions in AUC_0–24h_, C_max_ and C_trough_. The observed AUC_0–24_ and C_max_ of rifampicin were somewhat higher than previously reported values in patients with tuberculosis who used 10 mg/kg rifampicin.^27,40^ We used a limited sampling formula derived from a study in tuberculosis patients to estimate the AUC_0–24_ of rifampicin.^27^ It should be noted that this formula was not validated in patients with NTM disease, and this may have influenced the accuracy of the exposure estimation. The somewhat higher exposure to rifampicin is not expected to affect the extent of the interaction with minocycline, considering that the maximal inductive effect of rifampicin is largely achieved at a dose of 10 mg/kg.^26,41^

Notably, our interaction data align with previous reports on the rifampicin–doxycycline PK interaction,^18,19^ despite doxycycline being less subjected to metabolism.^33,37,42^ In patients with brucellosis, doxycycline AUC_0–12h_ and C_max_ were 58.1% and 31.2% lower, and clearance was 3-fold higher in 10 patients treated with doxycycline–rifampicin versus those treated with doxycycline–streptomycin.^18^ In addition, a study in hospitalized individuals aged 21–45 years reported a 54.5% reduction in AUC_0–24h_ and a 34.0% reduction in C_max_ of doxycycline, again with a 3-fold increased drug clearance.^19^ In future studies involving patients with MAC disease or other infections where co-administration of minocycline and rifampicin is considered (e.g. prosthetic joint infections),^15^ the evaluation of higher dosing of minocycline (e.g. 200 mg twice daily) may be considered to assess whether it can offset the observed PK interaction.

AEs were common, which is expected in a population with advanced disease receiving multidrug therapy alongside minocycline and rifampicin. Importantly, there were no severe AEs, and the majority were unrelated to minocycline. The most frequently reported minocycline-related AEs were gastrointestinal distress, vestibular disturbance and headache. No rare minocycline-induced AEs (e.g. serum sickness, lupus-like syndrome) were observed; however, our sample size was limited, and the study included two short courses of minocycline. No conclusions can be drawn regarding safety and tolerability in long-term use, as would be the case for MAC disease. Nevertheless, a recent meta-analysis reported no increase in serious AEs with minocycline use up to 2 years and found only a slightly elevated risk of withdrawal due to AEs compared to placebo (1.5, 95% CI 1.1–2.0).^16^

Whether the observed minocycline exposures are sufficient to attain pharmacokinetic–pharmacodynamic (PK–PD) targets for MAC disease is unknown. Given the lack of minocycline PK and PK–PD data in patients with MAC disease, an exploratory framework can be used to determine the AUC_0–24h_/MIC ratio by combining preclinical data with the observed AUC from the present study. While this allows a comparison with PK–PD targets established in a preclinical setting, all therapeutic interpretations remain exploratory. In a hollow fibre model simulating the PK–PD of minocycline monotherapy in MAC-PD, using MIC data of 55 clinical isolates from the Netherlands with an MIC50 of 8 mg/L, an AUC_0–24h_/MIC ratio of 45 was identified as necessary to maintain bacterial stasis, while a ratio of 59 was required to achieve a 1 log kill.^9^ Utilizing the MIC50 of 8 mg/L,^9^ our observed GM AUC_0–24h_ of 58.4 mg·h/L, and assuming 80% protein binding,^43^ the exploratory free AUC_0–24h_/MIC ratio of 1.5 falls short of these *in vitro* PK–PD targets. Applying the same conceptual framework to a minocycline- and rifampicin-containing regimen (i.e. MIC50 of 8 mg/L and our GM AUC_0–24h_ of 28.9 mg·h/L) results in an even lower exploratory AUC_0–24h_/MIC ratio of 0.7. However, it is important to consider that these *in vitro* PK–PD targets were established with minocycline monotherapy and have not been evaluated in a clinical setting. Furthermore, a subsequent hollow fibre study found that minocycline exposures in epithelial lining fluid, presumed to occur after a 200 mg once-daily dose, enhanced the bactericidal activity of an ethambutol–azithromycin regimen.^11^ Based on the results of the latter study, the evaluation of minocycline 200 mg daily as adjunctive therapy to azithromycin and ethambutol in MAC-PD may be considered for future studies.

This study had some limitations, most notably its small sample size and incomplete study participation. Only 11 participants completed the first study period, and nine participants were available for the evaluation of the effect of rifampicin on the PK of minocycline. Despite the small sample size, the observed interpatient variability of the pharmacokinetic parameters of minocycline without rifampicin co-administration corresponded with previous studies.^22,30^ Furthermore, the within-patient design of the study provided large statistical power to detect a PK drug interaction as sources of between-patient variability in PK are removed. Lastly, the reduction of total minocycline exposure during co-administration with rifampicin was consistent across the study population, which suggests the presence of a significant pharmacokinetic interaction. However, the magnitude of the effect and the clinical relevance of this interaction should be further investigated in future larger studies. An additional limitation, inherent to the fixed-order design, is the risk of time-dependent confounding. Specifically, changes in disease status due to the start of treatment may have influenced the PK of minocycline. In addition, although (adjustments to) concomitant medication and antimycobacterial drugs prior to and throughout the study period were critically reviewed for potential interactions with minocycline, the possibility of (unknown) interactions cannot be entirely excluded.

In conclusion, this study provides the first population PK estimates of minocycline in patients with NTM disease, offering a foundation for informed dosing strategies in future interventional trials, particularly for MAC disease. Co-administration with rifampicin resulted in a marked reduction in minocycline exposure of approximately 50%, likely due to induction of drug-metabolizing enzymes and/or transporters. The clinical relevance of this interaction and possible strategies to overcome its effects (e.g. dose adjustments of minocycline) should be evaluated in future studies.

## Supplementary Material

dkag262_Supplementary_Data

## Supplementary data

Tables S1 to S3 are available as Supplementary data at *JAC* Online.
