# Supplementary material for: Pharmacokinetics of minocycline with and without rifampicin co-administration in patients with nontuberculous mycobacterial disease
Source: J Antimicrob Chemother. 2026 Jul 30;81(8):dkag262. doi: 10.1093/jac/dkag262 (PMC13421885; doi:10.1093/jac/dkag262)
Supplement: dkag262_Supplementary_Data [file dkag262_supplementary_data.docx]

**Supplementary material**

Overview of content

Table S1. Overview of inclusion and exclusion criteria

Table S2. Validation parameters of the bioanalytical assays for minocycline and rifampicin

Table S3. Description of study withdrawals

Table S1. Overview of inclusion and exclusion criteria

| Inclusion criteria |
| --- |
| - *International guideline (ATS/ERS/ESCMIDIDSA) diagnostic criteria for NTM disease are met (1).* |
| - *The subject is eligible to start the guideline-recommended rifampicin-based regimen according to the treating physician (1, 2).* |
| - *Age ≥ 18 years.* |
| - *Signed and dated patient informed consent.* |
| Exclusion criteria |
| - *A relevant medical history or current condition that might interfere with drug absorption, distribution, metabolism or excretion (i.e. chronic gastro-intestinal disease, renal or hepatic disease).* |
| - *Diagnosed with cystic fibrosis (as this may affect the pharmacokinetics of drugs).* |
| - *Pregnant or breastfeeding (contra-indications for minocycline) or inadequate contraceptive measures (in view of the administration of rifampicin which interacts with oral contraceptive drugs, adequate contraceptive measures are abstinence from sexual activities and barrier methods).* |
| - *Use of drugs that cause a relevant drug interaction with minocycline, i.e. oral magnesium, , bismuth, aluminium, calcium, zinc or iron containing formulations, antacid drugs and drugs besides rifampicin that are strong inducers of metabolic enzymes, including barbiturates, carbamazepin and phenytoin (as judged by the investigators).* |
| - *ALAT > 3 times the upper limit of normal (normal <45 U/l).* |
| - *ASAT > 3 times the upper limit of normal (normal <35 U/l).* |
| - *An abnormal serum creatinine level (defined as a level that is higher than the upper limit of normal, i.e. >110 umol/l).* |
| - *Active alcohol abuse.* |
| - *Hypersensitivity to minocycline or to other tetracycline antibiotics.* |

Table S2. Validation parameters of the bioanalytical assays for minocycline and rifampicin

| Table S2. Validation parameters of the bioanalytical assays for minocycline and rifampicin | | | | | |
| --- | --- | --- | --- | --- | --- |
| Compound | Calibration range | Intraday | | Interday | |
|  | Concentration  (mg/L) | Accuracy  % | Precision  % | Accuracy  % | Precision  % |
| Minocycline | 0.025 - 20.0 | 89 - 103 | 1.5 - 2.8 | 91 - 102 | 0.0 - 2.3 |
| Rifampicin | 0.09 - 60.0 | 96 - 103 | 2.6 - 4.9 | 96 - 101 | 0.0 - 1.9 |

| 1 | Discontinued during the first minocycline course because of self-reported headache and nausea |
| --- | --- |
| 2 | Discontinued after the first minocycline course due to rifampicin-induced nausea and vomiting |
| 3 | Discontinued after the first minocycline course due to refusal to initiate rifampicin |

Table S3. Description of study withdrawals

References

1. Daley CL, Iaccarino JM, Lange C, Cambau E, Wallace RJ, Jr., Andrejak C, et al. Treatment of nontuberculous mycobacterial pulmonary disease: an official ATS/ERS/ESCMID/IDSA clinical practice guideline. Eur Respir J. 2020;56(1).

2. Haworth CS, Banks J, Capstick T, Fisher AJ, Gorsuch T, Laurenson IF, et al. British Thoracic Society guidelines for the management of non-tuberculous mycobacterial pulmonary disease (NTM-PD). Thorax. 2017;72(Suppl 2):ii1-ii64.
